# Ergodicity breaking from Rydberg clusters in a driven-dissipative many-body system

**DOI:** 10.1126/sciadv.adl5893

**Published:** 2024-03-01

**Authors:** Dongsheng Ding, Zhengyang Bai, Zongkai Liu, Baosen Shi, Guangcan Guo, Weibin Li, C. Stuart Adams

**Affiliations:** ^1^ Key Laboratory of Quantum Information, University of Science and Technology of China, Hefei, Anhui 230026, China.; ^2^ Synergetic Innovation Center of Quantum Information and Quantum Physics, University of Science and Technology of China, Hefei, Anhui 230026, China.; ^3^ State Key Laboratory of Precision Spectroscopy, East China Normal University, Shanghai 200062, China.; ^4^ School of Physics and Astronomy, and Centre for the Mathematics and Theoretical Physics of Quantum Non-equilibrium Systems, University of Nottingham, Nottingham NG7 2RD, United Kingdom.; ^5^ Department of Physics, Joint Quantum Centre (JQC) Durham-Newcastle, Durham University, South Road, Durham DH1 3LE, United Kingdom.

## Abstract

It is challenging to probe ergodicity breaking trends of a quantum many-body system when dissipation inevitably damages quantum coherence originated from coherent coupling and dispersive two-body interactions. Rydberg atoms provide a test bed to detect emergent exotic many-body phases and nonergodic dynamics where the strong Rydberg atom interaction competes with and overtakes dissipative effects even at room temperature. Here, we report experimental evidence of a transition from ergodic toward ergodic breaking dynamics in driven-dissipative Rydberg atomic gases. The broken ergodicity is featured by the long-time phase oscillation, which is attributed to the formation of Rydberg excitation clusters in limit cycle phases. The broken symmetry in the limit cycle is a direct manifestation of many-body collective effects, which is verified experimentally by tuning atomic densities. The reported result reveals that Rydberg many-body systems are a promising candidate to probe ergodicity breaking dynamics, such as limit cycles, and enable the benchmark of nonequilibrium phase transition.

## Teaser

Experimental evidence reveals collective effects in Rydberg gases, facilitating the study of nonequilibrium phase transitions.

## INTRODUCTION

Many-body systems typically relax to equilibrium due to ergodicity such that an observable becomes invariant with time (*1*–*3*). Often, the equilibration is so robust such that the observable quickly seeks fixed points in phase space after quenching control parameters, which is driven by Boltzmann’s ergodic (E) hypothesis (*4*). Exceptions, e.g., broken ergodicity due to symmetry breaking (*5*, *6*), have been extensively explored in integrable (*7*) and many-body localized systems (*8*, *9*). A recent example is the quantum many-body scars (*10*) of strongly interacting Rydberg atoms trapped in optical arrays (*11*), where nonergodic (NE) many-body dynamics take place in a constrained sub-Hilbert space (*12*, *13*). This leads to coherent revivals of the **Z**_2_ state (*14*), largely due to strong Rydberg atom interactions (*15*–*18*). In the presence of dissipation, it is common that quantum many-body coherence and entanglement are eliminated, leading to stationary states in long-time limit. The interplay between strong Rydberg interactions and dissipation, on the other hand, results to exotic nonequilibrium phenomena such as collective quantum jumps (*19*) and phase transitions (*20*–*28*). Optical bistability (*29*) and self-organized criticality (*30*, *31*) have been demonstrated experimentally.

**Fig. 1.**
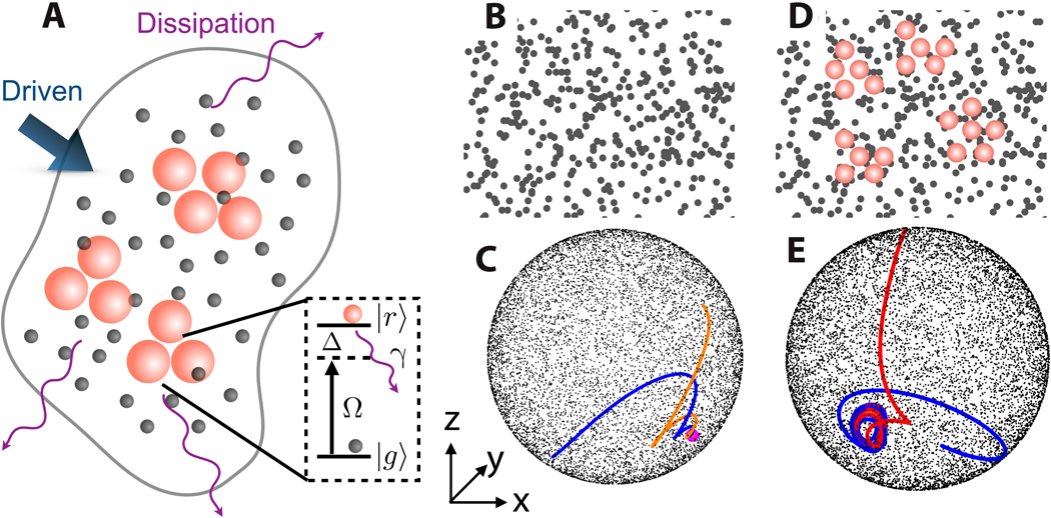
E and NE phases in a driven-dissipative Rydberg gas. (**A**) Atoms are laser excited from the ground state ∣*g*〉 (black sphere) to Rydberg state ∣*r*〉 (red, large sphere) with detuning Δ, Rabi frequency Ω, and decay rate γ (see inset for level diagram). Because of the strong Rydberg interaction, Rydberg excitations are separated in space, where the minimal distance is determined by the blockade radius. (**B**) E phase. When the Rydberg atom interaction is weak, the dissipation leads to homogeneous phase (a single fixed point). Black circles are the location of atoms. As shown in (**C**) any initial states (black dots) on or in the Bloch sphere will decay to the fixed point (magenta dot). Blue and orange curves depict trajectories of atoms from two different initial conditions. (**D**) NE phase. When the interaction is strong, closely packed Rydberg atoms (red circles) form clusters and are dynamically active, leading to nonstationary, collective dynamics. In phase space, the corresponding trajectories oscillate persistently, building up a limit cycle (**E**).

Here, we report observation of NE many-body dynamics in a thermal gas of strongly interacting Rydberg atoms. This setting, as depicted in Fig. 1A, is a dissipative many-body system of effective two-level atoms (ground and Rydberg states ∣*g*〉 and ∣*r*〉). Coherent laser coupling and strong long-ranged interactions in Rydberg state ∣*r*〉 compete with dissipation (Doppler and collisional effects, electronic state decay etc). A nonequilibrium phase transition is identified by quenching the detuning Δ, provided that the Rabi frequency Ω is above a critical value, in which a bifurcation between an E and weakly NE phase appears. The E phase corresponds to a weak interaction regime where the distribution of Rydberg atoms is homogeneous (Fig. 1B). Irrelevant to initial states, excitations of all atoms end at an identical fixed point in phase space (Fig. 1C). The NE phase features a nontrivial oscillation of the Rydberg population when the laser detuning is near resonant. Long-time many-body collective oscillations, in the order of milliseconds, are observed in the NE phase. The Rydberg population oscillations persist for a period that is much longer than any time scales of the relevant dissipation and laser Rabi frequency. Our analysis suggests that the broken ergodicity is induced by clustering of strongly interacting Rydberg atoms in free space (*32*–*35*), where dynamics of atoms in the thermal ensemble are synchronized and form the oscillatory phase, i.e., a limit cycle (Fig. 1, D and E) (*20*). The nonequilibrium dynamics is measured nondestructively through electromagnetically induced transparency (EIT). Because of the unprecedented level of controllability, thermal Rydberg atom vapor gases provide a platform to explore and probe nonergodicity of matter in addition to the Rydberg array simulator (*17*, *18*).

## RESULTS

### The experiment

We prepare rubidium-85 atom gases above room temperature (typically 45°C) with a density around 9.0 × 10^10^ cm^–3^. The atom is excited from ground state ∣*g*〉 to Rydberg state ∣*r*〉 through EIT, i.e., from ground state ∣*g*〉 = ∣5*S*_1/2_, *F* = 2〉 to intermediate state ∣*e*〉 = ∣5*P*_1/2_, *F*′ = 3〉 and then to Rydberg state ∣*r*〉 = ∣51*D*_3/2_〉 by a probe and coupling light field, respectively. The Rabi frequency and detuning of the probe and coupling light are denoted by Ω_p_ and Δ_c_. Both can be time dependent in our experiment. Here, a 795-nm laser is split into a probe beam and an identical reference beam by a beam displacer, which are both propagating in parallel through a heated Rb cell (10 cm long). The two-photon excitation with counter-propagating beams ensures that a narrow, low-velocity class of atoms are excited to Rydberg states due to velocity selections (*36*). The transmission signal of the probe beam is detected on a differencing photodetector as a transmission difference (*29*). Rydberg atoms vary for different realizations in thermal gases, such that conventional schemes based on electrically ionizing Rydberg atoms cannot be used here (*37*, *38*). Through EIT, however, the transmission signal (proportional to Rydberg atom populations) is measured continuously and dynamically while not demolishing atomic states (see Materials and Methods for details).

### Ergodic-breaking phase transition

**Fig. 2.**
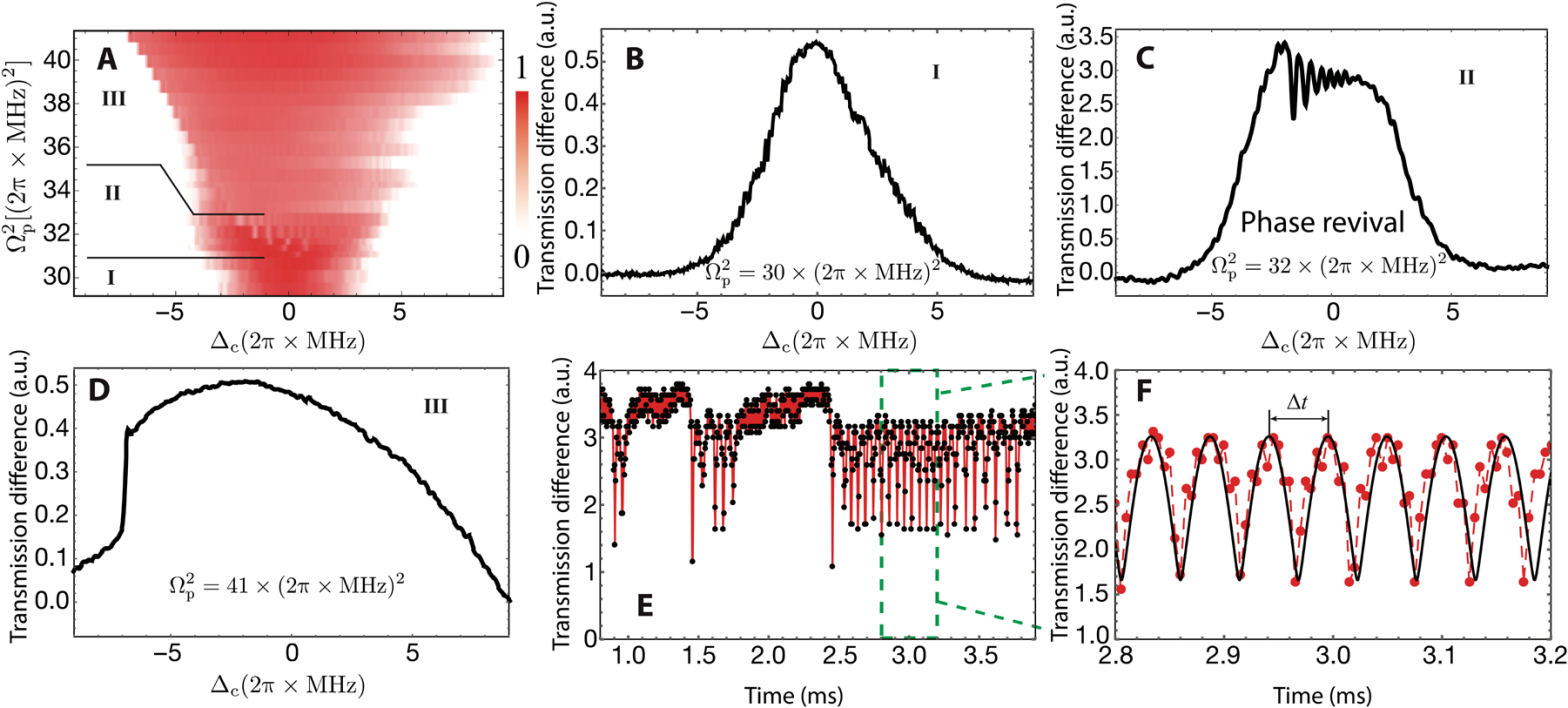
Nonequilibrium phase diagram and ergodicity breaking transition. (**A**) Transmission spectrum as a function of $\Omega_{p}^{2}$ and Δ_c_ with the sweep rate 2π × 6 MHz/ms. Three regions **I**, **II**, and **III** mark the E, ergodicity breaking, and strongly interacting phases, respectively. (**B**) Transmission spectrum when $\Omega_{p}^{2}=30\times {\left(2\pi \times \text{MHz}\right)}^{2}$ . This shows a typical profile in the region **I**. The acronym a.u. stands for the arbitrary unit. (**C**) The transmission spectrum when $\Omega_{p}^{2}=32\times {\left(2\pi \times \text{MHz}\right)}^{2}$ . When sweeping the detuning, transmission grows rapidly near the resonance. Then, the transmission quickly drops and starts to oscillate when further increasing the detuning. The oscillations mark the emergence of the nonequilibrium, E breaking phase. (**D**) In the strongly interacting phase, the transmission increases sharply at the critical point. Before and after the rapid change, the transmission changes smoothly when sweeping the detuning. (**E**) Observed time flow of the many-body NE state when stopping sweep Δ_c_ in the vicinity of the critical point, i.e., Δ_c_ ∼ −1.5 × 2π MHz and $\Omega_{p}^{2}=32\times {\left(2\pi \times \text{MHz}\right)}^{2}$ . Long time phase oscillations are found in the experiment. The black dots are experimental data, and the red line connects the experimental data to guide the eye. (**F**) Enlarged view of the region labeled in (E). The black curve is the fitting curve with function $\sqrt{1.64+1.60\text{cos}\left(1.88-116t\right)}+1.46$ . This fitting function is to guide the eye. The red dashed line connecting the data points shows visible difference between the experimental data and the fitted curve. This difference comes from the change of the oscillation frequency induced by the fluctuations of the system. The period of collapse and revival is Δ(*t*) = 0.053 ± 5.4 × 10^−6^ ms.

We probe the Rydberg population by changing the laser detuning Δ_c_ linearly from red to blue side while the probe laser is on resonance. Typically, Δ_c_ is quenched at a rate 2π × 6 MHz/ms. By raising the probe intensity, transmission against $\Omega_{p}^{2}$ and Δ_c_ is recorded and shown in Fig. 2A. The transmission is weak if the laser is away from the resonance. Close to the resonance, we observe that the transmission exhibits distinctive features depending on Ω_p_.

When the probe laser is weak $\Omega_{p}^{2}<30.9{\left(2\pi \times \text{MHz}\right)}^{2}$ , transmission is a smooth spectrum when increasing Δ_c_, corresponding to a dissipation dominant phase (region **I** in Fig. 2A; see also Fig. 2B). Increasing Ω_p_, a startling difference is that the transmission spectrum oscillates with increasing detuning, marked by region **II** in Fig. 2A. Specifically, the population of Rydberg atoms bifurcates from the E to NE phases, which takes place when $\Omega_{p}^{2}>30.9{\left(2\pi \times \text{MHz}\right)}^{2}$ . A typical example with $\Omega_{p}^{2}=32{\left(2\pi \times \text{MHz}\right)}^{2}$ is shown in Fig. 2C, where the transmission suddenly jumps from its maximal value when we increase Δ_c_. After passing the jump, the spectrum oscillates when further increasing Δ_c_. This nontrivial spectrum profile is unique in region **II**. When $\Omega_{p}^{2}$ is large (region **III** in Fig. 2A), the transmission increases sharply to the maximal value and then changes smoothly when increasing Δ_c_, shown in Fig. 2D. In a lattice, this corresponds to the antiferromagnetic phase (*20*). As our theoretical analysis shown in fig. S4, here, atoms end at different final states, while the overall transmission is stationary.

To further understand the dynamics, we stop sweeping Δ_c_ when the detuning is near the resonance and measure the evolution of the transmission. We observe a time flow of many-body collective collapsing and revival periodically as shown in Fig. 2 (E and F). The oscillation has a period of Δ*T* ≈ 0.053 ms, and a long lifetime of more than 1 ms, much longer than other characteristic time scales in the thermal Rydberg atom system.

### Ergodicity breaking from Rydberg atom clusters

The oscillation effect observed in Fig. 2 (E and F) corresponds to the broken ergodicity. This is induced by inhomogeneous Rydberg excitation in the thermal gas, resulting in spatial clusters formation of periodic oscillations, which violate the translational invariance (*3*, *35*) and ergodicity. We model the NE dynamics using a many-body Lindblad master equation $\dot{\rho }=L\left(\rho \right)$ , where the generator $\begin{array}{c} L\left(\cdot \right)=-i\left[\hat{H},(\cdot )\right]+\gamma \sum_{j}\left(J_{j}\left(\cdot \right)J_{j}^{†}-\frac{1}{2}\left\{J_{j}^{†}J_{j},(\cdot )\right\}\right) \end{array}$ with jump operator *J_j_* = ∣*g_j_*〉〈*r_j_*∣ (*19*, *29*), and Hamiltonian $\hat{H}$

$$\begin{array}{c} \hat{H}=\sum_{j}\left[-\Delta \left(t\right){\hat{n}}_{j}+\Omega \left(t\right){\hat{\sigma }}_{j}^{x}\right]+\sum_{j<k}V_{\mathrm{jk}}{\hat{n}}_{j}{\hat{n}}_{k} \end{array}$$ (1)

where *V_jk_* = *C*_6_/∣**R***_j_* − **R***_k_*∣^6^ (*C*_6_ to be the state-dependent dispersion coefficient) represents the van der Waals interaction between two Rydberg atoms locating at **R***_j_* and **R***_k_*. Operator ${\hat{\sigma }}_{j}^{x}=\left(|r_{j}\rangle \langle g_{j}|+|g_{j}\rangle \langle r_{j}|\right)/2$ flips the atomic state, and ${\hat{n}}_{j}=|r_{j}\rangle \langle r_{j}|$ is Rydberg density operator of the *j*-th atom. In the experiment, a large number of atoms is excited. To mimic this situation, we consider up to 10^3^ atoms in the simulation that are randomly distributed in three-dimensional space, such that spatial configurations are largely explored. We numerically solve dynamics of the atom ensemble with the discrete truncated Wigner method (*39*, *40*), which is suitable for dealing with interacting many-atom systems (*N* ≫ 1).

**Fig. 3.**
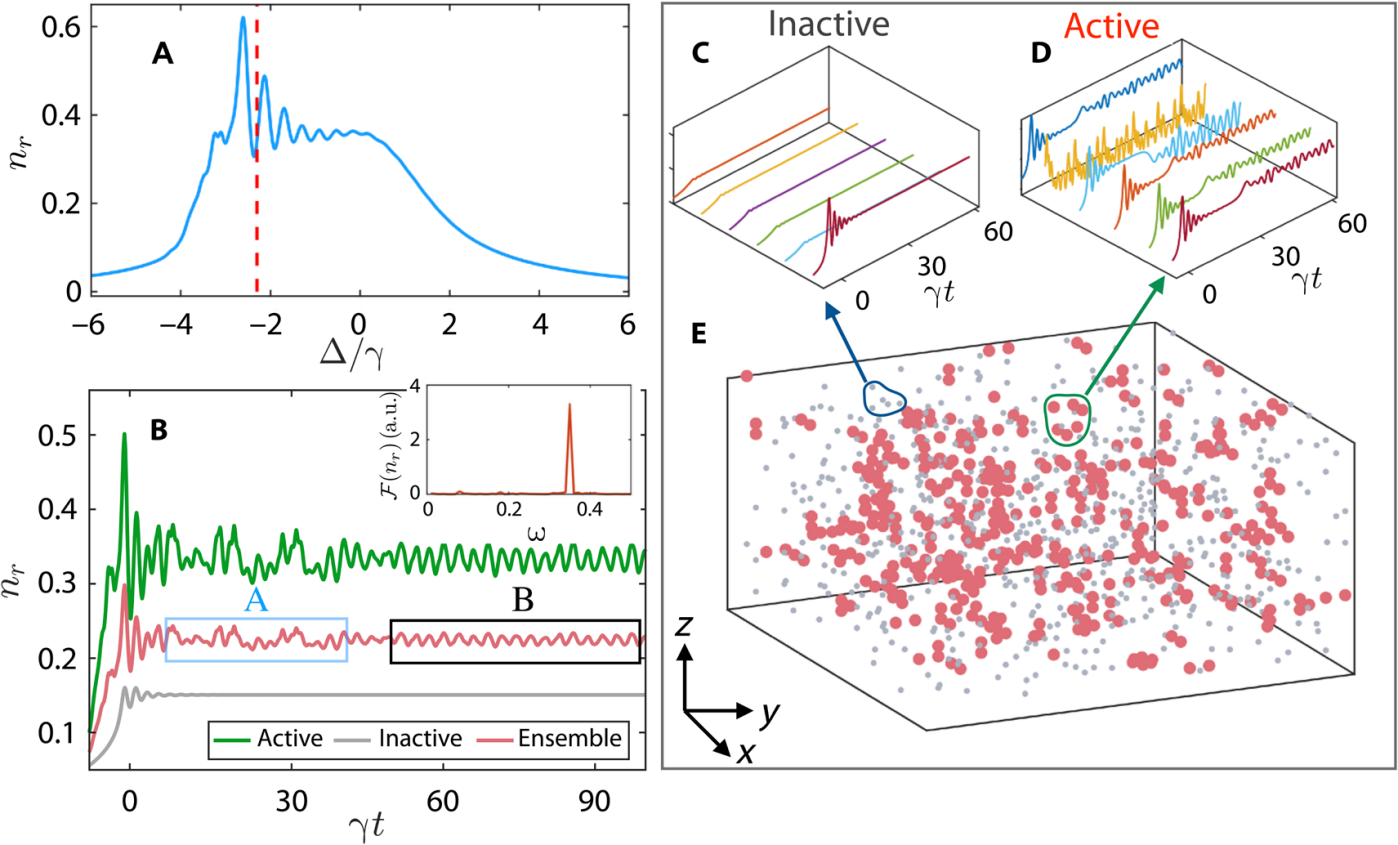
Rydberg clusters and synchronized oscillations. (**A**) Rydberg population *n_r_* as a function of Δ. When Δ approaches to the resonance, Rydberg population grows and starts to oscillate. (**B**) Dynamical oscillations of the active atoms (green) are synchronized at late time. The ensemble average (red), taking into account of contributions of the inactive atoms (gray), exhibits a collective oscillation (square box B) after a transient period (square box A). The frequency of the synchronized oscillation is shown in the inset panel. (**C**) When the quench is frozen at the detuning marked by the dashed line, it is found that most of atoms become dynamically inactive, where the population relaxes to equilibrium rapidly. (**D**) Dynamically active atoms, on the other hand, form spatial clusters whose population oscillates persistently. In (**E**), distributions of atoms in the ensemble are shown. The active and inactive atoms are denoted with red and grey spheres, respectively. At *t* = 0, Δ(0) = −10γ and atoms are in the ground state.

The optical transmission is linearly proportional to mean values $n_{r}=\sum_{j}\langle {\hat{n}}_{j}\rangle$ of the Rydberg population (please see text S1). As shown in Fig. 3A, the mean value *n_r_* resembles qualitatively the profile of the transmission spectrum (Fig. 3B) when sweeping the laser detuning. The high level of similarity permits us to gain insights from the numerical simulation into the observed dynamics. In (Fig. 3 C and D), examples of the Rydberg population are shown when detuning is frozen at Δ/γ = −2.35 (marked by the vertical line in Fig. 3A). It is found that dynamics of various atoms are largely different during the initial transient period, where the underlying trajectories of the population explore the phase space. At a later stage (γ*t* > 50), a notable fraction of atoms reaches a steady population and becomes dynamically inactive (see Fig. 3C). A sizable fraction of the atoms, on the other hand, is dynamically active, i.e., oscillating with time, as shown in Fig. 3D.

Unexpectedly, the global oscillations of different Rydberg atoms are synchronized at a later time, although oscillations show different amplitudes and frequencies at the early stage. In Fig. 3B, we plot *n_r_* corresponding to the data shown in Fig. 3 (C to E). After a transient period (square box A in Fig. 3B), the mean population stays in an oscillation phase. We find that oscillation frequencies of different atoms are locked and centered at a single frequency (inset of Fig. 3B). This synchronized oscillation resembles the oscillatory character observed in our experiment, as shown in Fig. 2E. We shall point out that the EIT setting allows to monitor transmission continuously, i.e., probing many-body dynamics without destroying Rydberg populations.

A closer look shows that the active atoms form spatial clusters. Here, the Rydberg level is shifted by the attractive van der Waals interaction of neighboring atoms, while Rydberg excitation happens under the antiblockade condition where the red detuned laser compensates the interaction rendered by multiple active atoms (*33*, *41*, *42*). Features of the Rydberg spatial distribution is then analyzed by using the Hopkins statistic (*43*), which gives a figure of merit of cluster tendency of the distribution. Our numerical calculations show that the Hopkins statistic is larger than 0.5 in the limit cycle phase, strongly suggesting the formation of spatial clusters. More details of the analysis can be found in fig. S5.

### Stability of the ergodic breaking phase

**Fig. 4.**
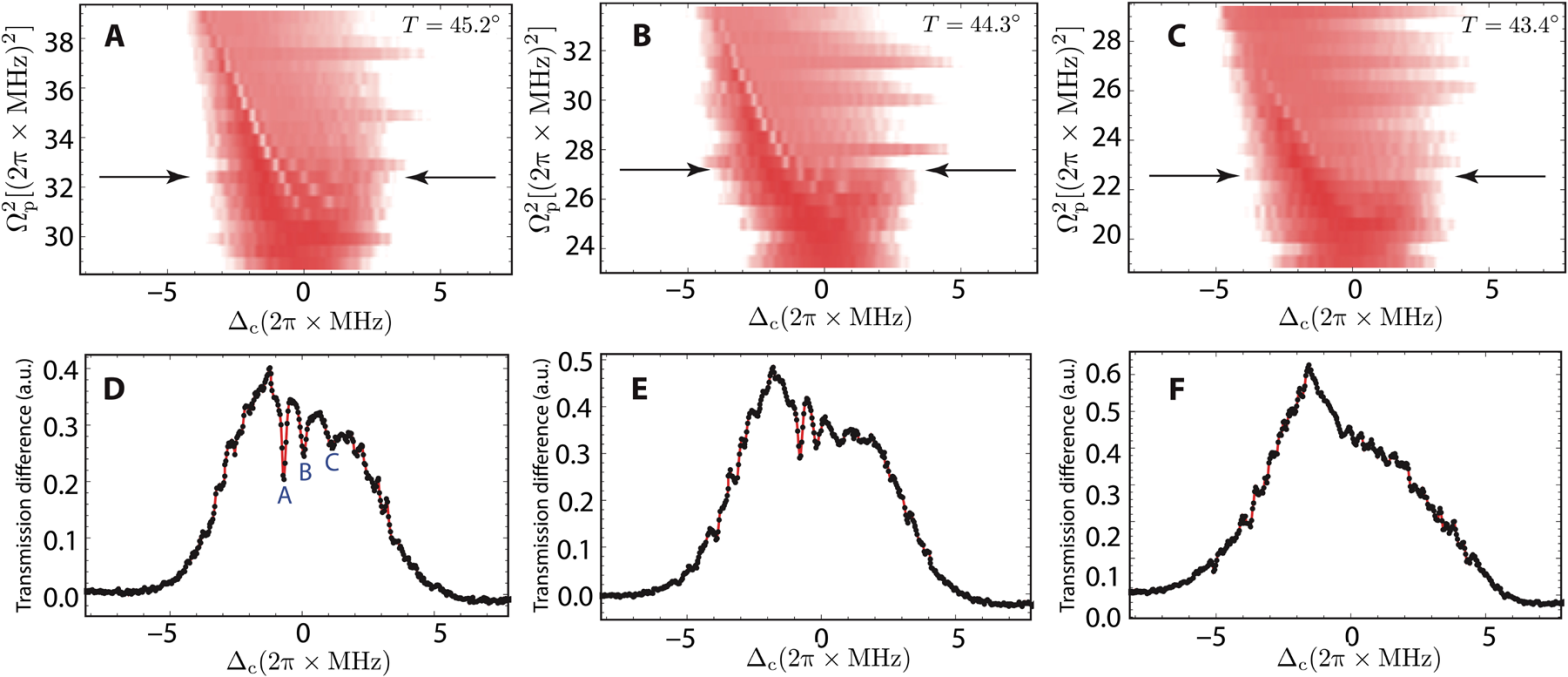
Density-dependent phase diagram. (**A** to **C**) Measured color map of the probe transmission against temperatures from *T* = 45.2°C to 44.3°C to 43.4°C, corresponding to the atomic densities of 9.52, 8.78, and 8.11 × 10^10^ cm^–3^. As the temperature increases, the oscillation amplitude becomes higher. (**D** to **F**) Oscillations of the probe transmission spectra at different temperatures. The data in (D) to (F) are the correspondence in (A) to (C) marked by the double arrows. The sweep rate is set as 2π × 11.4 MHz/ms.

The NE dynamics is stable against temperature and density. In our experiment, atomic densities can be varied by temperature of the gas. By decreasing the temperature *T* = 45.2°C, 44.3°C, and 43.4°C, we measure the probe transmission by sweeping Δ_c_ with a rate of 2π × 11.4 MHz/ms. As shown in Fig. 4A, oscillation regions are found in the transmission spectrum. The typical amplitudes of these oscillations are gradually faded out when we decrease the atomic density (see Fig. 4, A to C, for comparison].

We also extract the probe transmission data marked by the double arrows shown in Fig. 4 (D to F). In Fig. 4D, we use A, B, and C to indicate dips in the oscillations when sweeping the detuning. The dips are gradually disappeared when we decrease the atomic density, as seen in Fig. 4 (E and F). The reason is that average distances between Rydberg atoms are larger if atomic densities are low. This results to weaker Rydberg atom interactions, such that dissipation overrides the dynamics. If we decrease the atomic density further, then the phase transition would disappear completely and the probe transmission spectrum becomes smooth.The obtained density-dependent oscillations manifests strong many-body, collective characters in the nonequilibrium dynamics.

## DISCUSSION

We have studied the NE dynamics of nonequilibrium phase transition in a strongly interacting Rydberg gases. When the system approaches criticality, the Rydberg population is bifurcated into E and NE phases. In the vicinity of the critical point, the Rydberg population oscillated periodically for a long period of time. We have also observed the density-dependent, synchronized oscillation, which is a manifestation of the collective, many-body effect. The ergodicity breaking observed in our experiment is explained with the formation of Rydberg clusters. Early works have revealed the importance of inhomogeneous Rydberg excitation in the study of Rydberg soft matter, such as aggregates (*32*–*34*, *44*), bistability (*35*), and self-organization (*30*, *31*). Our study shows that clusters of Rydberg atoms trigger nonequilibrium, NE many-body dynamics despite the strong dissipation. Experimentally exploring broken ergodicity in a driven-dissipative Rydberg gas platform will expand the category of ergodicity of complex matter and nonequilibrium phenomenon (*45*, *46*), uncover the relation between the dissipation and the ergodicity (*47*), and find quantum technological applications (*48*, *49*).

## MATERIALS AND METHODS

### Experimental setup

Our experiment is carried out in a 10-cm-long glass cell with saturated pressure. We control the temperature to be 45°C (corresponding to the atomic densities of 9.0 × 10^10^ cm^–3^) in Fig. 2 and *T* = 45.2°C, 44.3°C, 43.4°C (corresponding to the atomic densities of 9.52, 8.78, and 8.11 × 10^10^ cm^−3^, respectively) in Fig. 4. In our experiment, three atomic levels (∣*g*〉 = ∣5*S*_1/2_, *F* = 2〉, ∣*e*〉 = ∣5*P*_1/2_, *F*′ = 3〉, and ∣*r*〉 = ∣51*D*_3/2_〉) of rubidium-85 are considered. Two laser beams, the probe light and the coupling light, couple the three level atoms and form a two photon excitation process via EIT, where the probe light (Toptica DL pro at 795 nm) couples energy levels ∣*g*〉 ↔ ∣*e*〉 and the coupling light (Toptica TA-SHG pro at 480 nm) couples energy levels ∣*e*〉 ↔ ∣*r*〉.

Before the 795-nm laser enters the rubidium vapor cell, it is split into the probe light (with waist of 1100 μm) and an identical reference light parallel to the probe light by a beam displacer. Both of the probe light and the reference beam transmit through the rubidium vapor cell, while the coupling light (with waist of 1000 μm) counter-propagates with the probe light and cover the probe light but not the reference light to reduce the Doppler effect. Last, the difference between the transmission signals of the probe and the reference beams are detected by a differential detector (Thorlabs, PB450A). The transmission spectral therefore is obtained via scanning the detuning of the coupling light Δ_c_ when the frequency of the probe light is locked to the atomic transition ∣*g*〉 ↔ ∣*e*〉. The phase diagrams (Figs. 2A and 4, A to C) are obtained via first sweeping the detuning Δ_c_ with rate 2π × 6 MHz/ms and then tuning the Rabi frequency of the probe light Ω_p_ by changing its power and again sweeping the detuning Δ_c_ for the next Rabi frequency Ω_p_.

## Supplementary Materials

### This PDF file includes:

Texts S1 to S3 Figs. S1 to S8 References
