# Supplementary material for: Ergodicity breaking from Rydberg clusters in a driven-dissipative many-body system
Source: Sci Adv. 2024 Mar 1;10(9):eadl5893. doi: 10.1126/sciadv.adl5893 (PMC10911772; doi:10.1126/sciadv.adl5893)
Supplement: Supplementary file 1 — Texts S1 to S3 Figs. S1 to S8 References [file sciadv.adl5893_sm.pdf]

Science Advances
AAAS

Supplementary Materials for

Ergodicity breaking from Rydberg clusters in a driven-dissipative many-body system

Dongsheng Ding *et al.*

Corresponding author: Dongsheng Ding, dds@ustc.edu.cn; Baosen Shi, drshi@ustc.edu.cn;
Weibin Li, weibin.li@nottingham.ac.uk, C. Stuart Adams, c.s.adams@durham.ac.uk

Sci. Adv. **10**, eadl5893 (2024)
DOI: 10.1126/sciadv.adl5893

This PDF file includes:

Texts S1 to S3
Figs. S1 to S8
References

Supplementary Text

In this supplementary material we give more details on the experiment and theoretical analysis.

1 Transmission of the probe laser

In this section, we will derive the relation between transmission of probe field and Rydberg population of the three level system. Electric field of the laser fields interacting with the atomic gas reads $\mathbf{E}(\mathbf{r},t) = \mathbf{E}_p + \mathbf{E}_c = \sum_{l=p,c} \mathbf{e}_l \mathcal{E}_l \exp[i(\mathbf{k}_l \cdot \mathbf{r} - \omega_l t)] + \text{c.c.}$, where $\mathbf{e}_l$ ($\mathbf{k}_l$) is the unit polarization vector (wavevector) of the electric-field component with the envelope with $\mathcal{E}_l$ $(l = p, c)$. Here, probe laser field $\mathbf{E}_p$ (with wave number $k_p = 2\pi/\lambda_p$, wavelength $\lambda_p = 780\,\text{nm}$, and Rabi frequency Ω_p) couples to the transition between the ground state $|g\rangle$ and the excited state $|e\rangle$; strong, continuous-wave control laser field $\mathbf{E}_c$ (with wave number $k_c = 2\pi/\lambda_c$, wavelength $\lambda_c = 480\,\text{nm}$, and Rabi frequency Ω_c) couples to the transition between state $|e\rangle$ and the Rydberg $|r\rangle$. Δ_p and Δ_c are the detuning of the probe and control field; The probe and control laser fields counter-propagate in the Rydberg gas [i.e., $\mathbf{k}_p = (0, 0, -k_p), \mathbf{k}_c = (0, 0, k_c)$]. Once excited to the Rydberg state, the interaction between $\left|51D_{3/2}\right\rangle$ is attractive with dispersive coefficient $C_6 < 0$ [see Fig. S1]. By including the interatomic coupling V_{jk} between the excited

Rydberg atoms, the Hamiltonian in the interaction picture and rotating-wave approximation reads ($\hbar = 1$),

$$\hat{H}_{3LS} = \sum_{j=1}^{N} \left[-\tilde{\Delta}_p \hat{\sigma}_{ee}^j - (\tilde{\Delta}_p + \tilde{\Delta}_c)\hat{\sigma}_{rr}^j - \left(\Omega_p \sigma_{eg}^j + \Omega_c \sigma_{re}^j + \text{H.c.}\right) + \sum_{j<k} V_{jk} \sigma_{rr}^j \sigma_{rr}^k \right],$$

where $\sigma_{\alpha\beta}^j = |\alpha_j\rangle\langle\beta_j|$ ($\alpha, \beta = e, g, r$), the velocity distribution of the atoms via their Doppler shift for each transition are included with $\tilde{\Delta}_p = \Delta_p - k_p v$ and $\tilde{\Delta}_c = \Delta_c + k_c v$. We then define a "collective operator" valid for dense atomic gases. For a small volume $\Delta V(\mathbf{r})$ centered around $\mathbf{r}$, there are ΔN_r atoms. The collective spin operator for these atoms is $\hat{\sigma}_{\nu\mu}(\mathbf{r}) = \frac{1}{\Delta N_{\mathbf{r}}} \sum_{j \in \Delta V(\mathbf{r})} \hat{\sigma}_{\nu\mu}^j e^{i\mathbf{k}\mathbf{r}_j}$. In the mean field (MF) approximation by neglecting two-body correlations, we obtain the optical Bloch equation of expectation values of operators $\rho_{\alpha\beta} \equiv \langle \hat{\sigma}_{\alpha\beta} \rangle$,

$$\frac{\partial}{\partial t}\rho_{gg} = \Gamma_{ge}\rho_{ee} - i\frac{\Omega_p}{2}\rho_{eg} + i\frac{\Omega_p^*}{2}\rho_{ge}, \tag{S1a}$$
$$\frac{\partial}{\partial t}\rho_{ee} = \Gamma_{er}\rho_{rr} - \Gamma_{ge}\rho_{ee} + i\frac{\Omega_p}{2}\rho_{eg} - i\frac{\Omega_p^*}{2}\rho_{ge} - i\frac{\Omega_c}{2}\rho_{re} + i\frac{\Omega_c^*}{2}\rho_{er}, \tag{S1b}$$
$$\frac{\partial}{\partial t}\rho_{rr} = -\Gamma_{er}\rho_{rr} + i\frac{\Omega_c}{2}\rho_{re} - i\frac{\Omega_c^*}{2}\rho_{er}, \tag{S1c}$$
$$\frac{\partial}{\partial t}\rho_{ge} = id_{ge}\rho_{ge} - i\frac{\Omega_p}{2}(\rho_{ee} - \rho_{gg}) + i\frac{\Omega_c^*}{2}\rho_{gr}, \tag{S1d}$$
$$\frac{\partial}{\partial t}\rho_{gr} = id_{gr}\rho_{gr} - i\frac{\Omega_p}{2}\rho_{er} + i\frac{\Omega_c}{2}\rho_{ge} - iV_{\text{MF}}\rho_{rr}\rho_{gr}, \tag{S1e}$$
$$\frac{\partial}{\partial t}\rho_{er} = id_{er}\rho_{er} - i\frac{\Omega_p^*}{2}\rho_{gr} - i\frac{\Omega_c}{2}(\rho_{rr} - \rho_{ee}) - iV_{\text{MF}}\rho_{rr}\rho_{er}, \tag{S1f}$$

where V_{MF} is the MF shift. We have defined $d_{\alpha\beta} = \Delta_\alpha - \Delta_\beta + i\gamma_{\alpha\beta}$ ($\alpha \neq \beta$), with $\Delta_e = \tilde{\Delta}_p$, $\Delta_r = \tilde{\Delta}_p + \tilde{\Delta}_c$, and $\gamma_{\alpha\beta} \equiv (\Gamma_\alpha + \Gamma_\beta)/2 + \gamma_{\alpha\beta}^{\text{col}}$. Here $\Gamma_\beta \equiv \sum_{\alpha<\beta} \Gamma_{\alpha\beta}$ with $\Gamma_{\alpha\beta}$ the spontaneous emission decay rate, and $\gamma_{\alpha\beta}^{\text{col}}$ the dephasing rate between $|\alpha\rangle$ and $|\beta\rangle$. By solving Eqs. (S1d) and (S1f) adiabatically (assuming ground state population $\rho_{gg} \approx 1$), we can obtain the stationary solution of coherence ρ_{ge},

$$\rho_{ge} \simeq \frac{1}{\tilde{\Delta}_p^2 + \Gamma_{ge}^2/4}\left(\frac{\tilde{\Delta}_p\Omega_p}{2} - \frac{|\Omega_c|^2\Gamma_{ge}}{4\Omega_p^*}\rho_{rr}\right) + \frac{i}{\tilde{\Delta}_p^2 + \Gamma_{ge}^2/4}\left(\frac{\Gamma_{ge}\Omega_p}{4} + \frac{|\Omega_c|^2\tilde{\Delta}_p}{2\Omega_p^*}\rho_{rr}\right) \tag{S2}$$

Here we focus on the transmission information of the probe field, thus the propagation of probe field should be taken into account. Applying the continuous density approximation, this yields the one dimensional (1D) Maxwell equations,

$$i\left(\frac{\partial}{\partial z}+\frac{1}{c}\frac{\partial}{\partial t}\right)\Omega_p(z)+\frac{k_p}{2}\chi_p(z)\Omega_p(z)=0, \tag{S3}$$

where $\chi(z) = 2\mathcal{N}|\mu_{eg}|^2 \int dv f(v)\rho_{ge}(z,v)/[\varepsilon_0\Omega_p]$ is the integrated susceptibility with atomic density $\mathcal{N}$ and dipole moment μ_{eg} between the ground state and excited state. $f(v) = 1/(\sqrt{\pi}v_T)\exp[-(v/v_T)^2]$ is the Maxwell-Boltzmann velocity distribution. $v_T = \sqrt{2k_BT/M}$ is the thermal velocity (M mass of Rb atoms). We can derive the transmission of the probe field $T^{3LS} = \exp[-k_p\mathrm{Im}(\chi_p)L]$ with medium length L. In our experiment, the measured transmission T is the difference between T^{3LS} and the reference transmission (i.e., transmission without the control field $T^{2LS} = T^{3LS}|_{\Omega_c=0}$). By substituting the solution (S2) into the transmission formula, then we can obtain,

$$\begin{aligned} T &= T^{3LS}-T^{2LS} \\ &\simeq \exp\left(-\int dv f(v)\frac{2k_p\mathcal{N}|\mu_{eg}|^2\Gamma_{ge}}{\varepsilon_0\hbar(4\tilde{\Delta}_p^2+\Gamma_{ge}^2)}L\right)\times\int dv f(v)\frac{2k_p\mathcal{N}|\mu_{eg}|^2|\Omega_c|^2\Gamma_{ge}L}{\varepsilon_0\hbar(4\tilde{\Delta}_p^2+\Gamma_{ge}^2)|\Omega_p(0)|^2}\rho_{rr}(v). \end{aligned} \tag{S4}$$

This expression is valid when the Rydberg excitation is small, which is the case in our experiment. In the experiment atoms within a narrow range of velocity group are excited (*36*), which indicates that the Rydberg population is approximately independent of the velocity, i.e. $\rho_{rr}(v) \approx \rho_{rr}$. From Eq. (S4), we can see that the transmission T is proportional to the Rydberg excitation ρ_{rr}.

2 Theoretical Model of the many-body driven-dissipative dynamics

2.1 Quantum Master equation

The dynamics of the Rydberg atom can be described by an effective two-level model (*29, 30*). We consider an ensemble of N two-level atoms, where the electronic ground state $|g\rangle$ is coupled to excited state $|r\rangle$ by laser fields with an effective, time-dependent Rabi frequency $\Omega(t)$ and detuning $\Delta(t)$. Atoms in state $|r\rangle$ interact strongly through van der waals interaction $V_{jk} = C_6/|\mathbf{R}_j - \mathbf{R}_k|^6$ with C_6 and $\mathbf{R}_{j(k)}$ the dispersion coefficient and location of the $j(k)$-th atom. The Hamiltonian $\hat{H}$ of the many-body system reads ($\hbar \equiv 1$),

$$\hat{H} \quad = \sum_j \left[-\Delta(t)\hat{n}_j + \Omega(t)\hat{\sigma}_j^x\right] + \sum_{j<k} V_{jk}\hat{n}_j\hat{n}_k, \tag{S5}$$

where $\hat{\sigma}_j^x = (|r_j\rangle\langle g_j| + |g_j\rangle\langle r_j|)/2$ flips the atomic state and $\hat{n}_j = |r_j\rangle\langle r_j|$ is the projection operator of the excited state. Including the dissipation, dynamics of the system density matrix ρ is modelled by a Lindblad master equation,

$$\dot{\rho}(t) = \mathcal{L}\rho(t), \tag{S6}$$

where $\mathcal{L}(\cdot) = -i[\hat{H}, (\cdot)] + \gamma \sum_j (J_j(\cdot)J_j^\dagger - \frac{1}{2}\{J_j^\dagger J_j, (\cdot)\})$ with $J_j = |g_j\rangle\langle r_j|$ is the jump operator (*19, 29*) and γ is the effective decay rate.

2.2 Mean field approach

For small systems (i.e., about 10 atoms), the quantum master equation can be solved numerically. However, the Hilbert space of the Hamiltonian grows with 2^N, while the dimension of the density matrix is 2^{2N}. The computational complexity prevents us from numerically solving the many-body problem when $N > 10$ with typical computers. Due to the dissipation, many-body correlations may be weak, such that we could employ approximations, such as the mean-field (MF) theory and truncated discrete Wigner method to simulate the dynamics.

In the MF approach, the many-body density matrix ρ is decoupled into tensor products of individual ones, $\hat{\rho} \approx \Pi_i \, \hat{\rho}_i$. This decoupling essentially ignores correlations between different sites (*50*). This is a good approximation in a three-dimensional system with large number of atoms (typically $10^3 \sim 10^4$ Rydberg atoms are prepared in the experiment). In the MF calculation, mean values of spin operators $s_j^\mu = \langle \hat{\sigma}_j^\mu \rangle$ ($\mu = x, y, z$) are calculated, whose dynamics is governed by the following equations of motion,

$$\frac{ds_j^x}{dt} = -\Delta s_j^y - \frac{\gamma}{2} s_j^x + \sum_{j<k} V_{jk} s_j^y n_r^k, \tag{S7a}$$

$$\frac{ds_j^y}{dt} = \Delta s_j^x - \frac{\gamma}{2} s_j^y - \Omega s_j^z - \sum_{j<k} V_{jk} s_j^x n_r^k, \tag{S7b}$$

$$\frac{ds_j^z}{dt} = \Omega s_j^y + \frac{\gamma}{2}(1 - 2s_j^z), \tag{S7c}$$

where $n_r^j = 0.5 - s_j^z$ represent the Rydberg population on site $\mathbf{R}_j$.

2.3 Mean field phase diagram

The limit cycle and uniform phase can already be revealed from the MF theory. We first investigate a bipartite (i.e. two sites labelled by A and B) subspace that represents the minimal model of our setting. In this case, Eq. (S7) is simplified to the following coupled equations of the $A - B$ subspace,

$$\frac{ds_A^x}{dt} = -\Delta s_A^y - \frac{\gamma}{2} s_A^x + V_{AB} s_A^y n_r^B, \tag{S8a}$$

$$\frac{ds_A^y}{dt} = \Delta s_A^x - \frac{\gamma}{2} s_A^y - \Omega s_A^z - V_{AB} s_A^x n_r^B, \tag{S8b}$$

$$\frac{ds_A^z}{dt} = \Omega s_A^y + \frac{\gamma}{2}(1 - 2s_A^z), \tag{S8c}$$

Equations for B site can be obtained by swapping index A and B in Eq. (S8). The fixed points of Eqs. (S8) can be found by setting $\dot{s}_{A(B)}^x = \dot{s}_{A(B)}^y = \dot{s}_{A(B)}^z = 0$. Two types of fixed points are found: a uniform phase (i.e., $s_z^A = s_z^B$) correspond to spatially homogeneous excitation, and a

nonuniform one (i.e., $s_z^A \neq s_z^B$) could contribute to both the antiferromagnetic and oscillatory phases. We further analyze the linear stability of the fixed points by calculating eigenvalues λ_j of the Jacobian matrix of Eqns. (S8) (*51*). If the real parts of all eigenvalues are negative, the corresponding solution is stable; otherwise, it is unstable. MF phase diagrams of the bipartite subspace are shown in Fig. (S2)(a). We find that the system is dominated by three cases: uniform (UNI), antiferromagnetic (AF), and oscillatory (OSC). The latter two correspond to nonuniform distribution.

Dynamically one can probe the many-body MF phases by sweeping the laser detuning Δ from red to blue side linearly (i.e., $\Delta(t) = \Delta_0 + a \times t$). When the detuning Δ approaches the resonance, the population of Rydberg atoms bifurcates from the UNI phase to the oscillatory phase. The latter is found when $\Omega > 0.4$ [Fig. (S2)(b)].

Fig. S3 shows the dynamics of the Rydberg populations with different Ω. When Ω is small, the steady state is the UNI phase [Fig. S3(a1)] where excitation of atoms are identical, then changes to the OSC phase [Fig. S3(b1)] and then to the AF phase by increasing Ω [Fig. S3(c1)]. For UNI phase, atoms decay to a single fixed point (blue dot) [Fig. S3(a2)]. These unstable nonuniform fixed points lead to limit cycles [Fig. S3(b2)], in which the Rydberg population oscillates periodically in time [Fig. S3(b3)]. Further increasing Ω, the nonuniform phase becomes stable which means the system shows an antiferromagnetic pattern [Fig. S3(c1)]. Their phase space trajectories can decay to two different fixed points [Fig. S3(c2)]. In both the uniform and antiferromagnetic phase, the population dynamics is stationary, which is in sharp contrast to the population dynamics found in the oscillatory phase.

2.4 Discrete truncated Wigner approximation

The number of Rydberg excitation is large (i.e., $10^3 \sim 10^4$) in the experiment. To simulate dynamics of the Rydberg atoms, we consider an ensemble of N atoms that are randomly dis-

tributed in space [see Fig. 3(b3) in the main text]. To efficiently characterize the quantum many-body behaviour of such system, we apply the discrete truncated Wigner approximation (DTWA; beyond MF phase space method), where the interaction $\hat{n}_j \otimes \sum_k \hat{n}_k$ is replaced with the MF term $\hat{n}_j \sum_k \langle \hat{n}_k \rangle$, and the quantum fluctuations are incorporated in initial states by a Wigner distribution (*39*).

In the DTWA method, we describe the initial state by a Wigner probability distribution, $p^k_{\mu,a_\mu}(\mu = x, y, z$; the subscript a_μ denotes the index of each trajectory, k denotes the position of Rydberg atom) for certain discrete configurations of Bloch vector elements, s^μ_j $(\mu = x, y, z)$. Consider the eigen-expansion of the spin operators, $\hat{\sigma}^\mu_k = \sum_{a_\mu} \eta^k_{\mu,a_\mu} |\eta^k_{\mu,a_\mu}\rangle\langle\eta^k_{\mu,a_\mu}|$, where η^k_{μ,a_μ} and $|\eta^k_{\mu,a_\mu}\rangle$ denote the eigenvalues and eigen-vectors, respectively. Then, we select the "a-th" eigenvalue, $\lambda^k_\mu(t = 0) = \eta^k_{\mu,a_\mu}/2$, with probability $p^k_{\mu,a_\mu} = \mathrm{Tr}[\hat{\rho}^k_0 |\eta^k_{\mu,a_\mu}\rangle\langle\eta^k_{\mu,a_\mu}|]$. All the atoms initially populate in the ground state $|g\rangle$, with density matrix $\hat{\rho}^k_0 = |g\rangle\langle g|$, which leads to fixed classical spin component along z, $s^k_z = -1/2$, and fluctuating spin components in the orthogonal directions $s^k_{x(y)} \in \{-1/2, 1/2\}$, each with 50% probability. Mean values of observable (i.e., the Rydberg population) are calculated by averaging over the ensemble. In the simulation, we test the robustness of the oscillatory phase against the quantum fluctuations. The DTWA result is shown in Fig. 3 of the main text. The numerical simulation shows that even though fluctuations exist, the oscillation phase keeps its shape in the dynamics process.

We have shown the dynamical evolution of the Rydberg population in Fig. 3(b1)-(b2) in the main text. Based on the dynamical behavior, the atomic system can be distinguished into two components: Most of the atoms reaches a steady population and becomes dynamically inactive (N_{in} atoms labelled with small gray circles), and another one is non-stationary (N_{active} atoms labelled with large red circles). We label atoms with the non-stationary solutions as dynamically active atoms. The active atoms form spatial clusters in the Rydberg gas and give rise to the oscillatory phase into the ensemble.

For random gases, we can use the ratio N_{active}/N to character active fraction of the system. Here the ratio $N_{\text{active}}/N \to 1$ means that most of the atoms is dynamically active, and $N_{\text{active}}/N \to 0$ denotes the system remains in the inactive phase. Examples of DTWA phase diagrams (with order parameter N_{active}/N) are shown in Fig. S4(a). It is found that the active phase emerges in the black curve marked region where $N_{\text{active}}/N > 0.4$, which has a similar shape as the experimental phase diagram Fig. 2(a).

2.5 Hopkins statistic

In the strong interaction regime, active atoms form Rydberg clusters, which violates the translation invariance and induces the broken ergodicity in free space. To identify the formation of Rydberg clusters, we calculate the Hopkins statistic (*43*). The strategy of Hopkins test statistics is the follows: We randomly sample M test points [i.e., $\mathbf{X}_t = (x_j^t, y_j^t, z_j^t)$, $j = 1, 2, ..M$] in our simulation space (see blue dots in Fig. S5). The Hopkins statistics requires $M \ll N_{\text{active}}$. Thus, we set $M = 10\% N_{\text{active}}$. As demonstrated above, the position of active atom can be identified with $\mathbf{X}_a = (x_j^a, y_j^a, z_j^a)$ $(j = 1, 2, ..N_{\text{active}})$ (see red dots in Fig. S5). Two types of distances can be defined: (i) The minimum distance $u_j = \min(U_j)$ is found from $\mathbf{X}_t$ to its nearest site in active ensemble $\mathbf{X}_a$; (ii) We can also calculate the minimum distance $w_j = \min(W_j)$ from a randomly selected site in $\mathbf{X}_a$ to its nearest neighbor (M out of the available N_{active} sites are marked randomly with green circle in Fig. S5). The Hopkins statistic in our scheme is defined as,

$$H = \frac{\sum_{j=1}^{M} u_j}{\sum_{j=1}^{M} u_j + \sum_{j=1}^{M} w_j}. \tag{S9}$$

The values of H are calculated by averaging over many atoms (e.g., $N_{\text{t}} = 1000$) until the results converge well. This statistic compares the nearest-neighbor distribution of randomly selected locations to that for the randomly selected sites. $H = 0.5$ reflects the random distribution of atoms, and the Rydberg atoms are aggregated or clustered when $H > 0.5$ (*43*).

The Hopkins statistic of the atoms are shown in Fig. S4(b). It is clear to observe that: (i) In the ergodic phase, $H \simeq 0.5$ and the atomic ensemble is homogeneous; (ii) In the non-ergodic/oscillatory phase (marked by the black curve), $H \simeq 0.7$. The latter regime perfectly overlaps with the dynamically active phase, in which the Rydberg clusters form. (iii) In a stationary cluster (SC) phase, $H \simeq 0.9$. In this regime, the number of active atom decreases, but most of inactive atoms decays to different fixed points. It results in multistable stationary phases (*29, 30*).

We present three examples of the dynamical evolution in Fig. S6, covering the E-, NE-, and SC-phases. In the E-phase, the mean value of the Rydberg population n_r is small [Fig. S6(a1)]. All atoms evolve into uniform fixed points with weak fluctuations [Fig. S6(a2)]. In the NE-phase, a large fraction of the atom is dynamically active. At the same time, due to the strong interaction, most of the inactive atoms play a minor role in contributing to the Rydberg population (also see Fig. 3(c) in the main text). Here n_r oscillates periodically at late time [Fig. S6(b1)-(b2)]. In the SC phase, the number of active atoms decreases. Although clusters are found in this phase, the overall dynamics is time independent, as the number of active atoms is small.

3 The discussion on oscillation frequency

Further experiment analysis has been carried out to identify the oscillation period in the experiment. Using the optical transmission in the oscillation regime, we extracted a mean oscillation frequency from the experimental data, shown in Fig. S7, we find that the transmission oscillates faster as the power of probe field Ω_p^2 increases. At a given laser power, the oscillation frequency increases when we decrease the temperature (inset of Fig. S7). To understand the temperature and power dependence, we note that $\gamma_{gr}^{\rm col} = \mathcal{N} v_T \sigma$ with atomic density $\mathcal{N}$ and σ the collisional cross-section between ground- and Rydberg state. The number density of the atomic gas also depends on the temperature (*52*). By scaling the laser power with $\gamma = \gamma_{gr}^{\rm col}$, we find that these

data almost collapse to a single curve. Note that in the experiment, there are other sources of dissipations, which have not been taken into account in the above scaling.

We also calculate the oscillation frequency numerically based on the DTWA simulation to verify our experimental data. For fixing $\Delta = -2.4\gamma$ and $C_6 = -8\gamma R_0$ (with average atomic distance R_0), we find that the oscillation frequency increase with Ω^2 [see Fig. S8(a)]. This increase is not continuous but experiences abrupt changes with narrow plateaus, where the frequency is stable along the plateaus. The trend of experimental results is captured well by our numerical simulation. We furthermore calculate the oscillation frequency with interaction strength C_6. The result is shown in Fig. S8(b). We find that the oscillation frequency exhibits a sequence of plateaus when C_6 increases continuously.

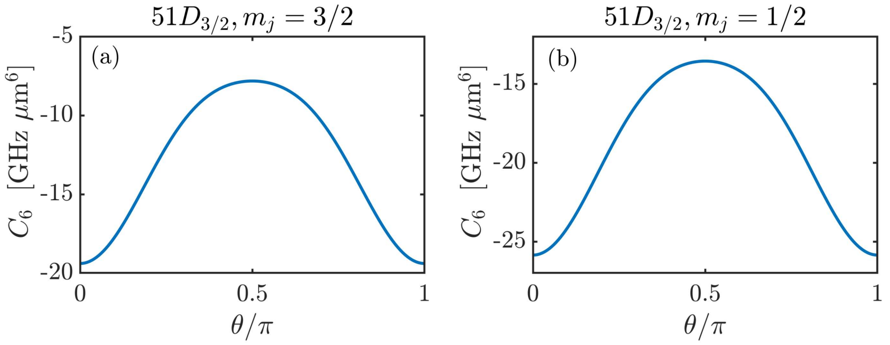

Figure S1: Angular dependent dispersion coefficient $\tilde{C}_6(\theta)$ in states $|51D_{3/2}\rangle$. Mean value C_6 is used in the numerical simulations.

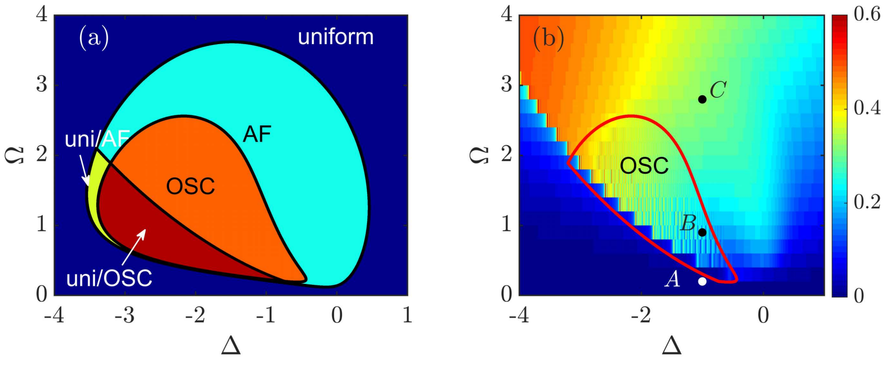

Figure S2: MF phase diagrams. (a) The phase diagram is mainly occupied by the UNI, AF, and OSC phase. (b) The many-body phases could be detected by sweeping detuning Δ from red to blue side linearly (i.e., $\Delta(t) = \Delta_0 + a \times t$). The Rydberg population $n_r(t)$ in the $\Delta - \Omega$ plane is sketched. The oscillatory phase can be identified by Rydberg excitation. In the calculation, we set $V_{AB} = -8$, $\gamma = 0.5$ and $a = 0.01$.

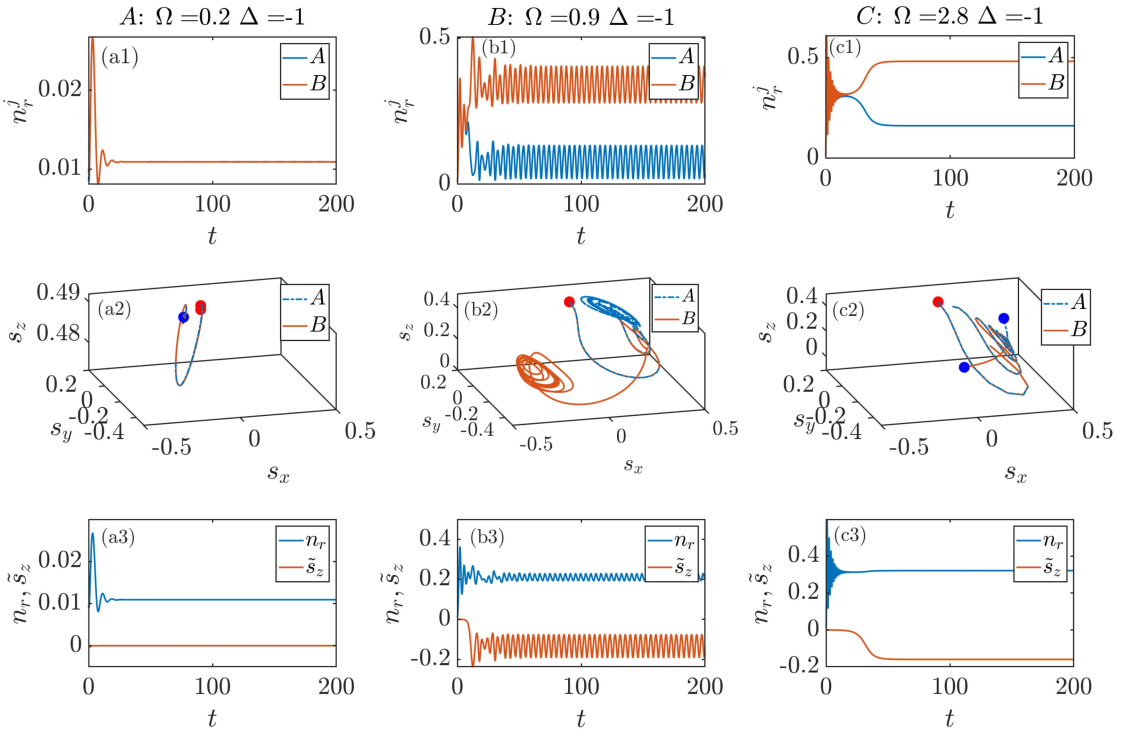

Figure S3: **Excitation of different phases.** (a1) Rydberg populations n_r^j $(j = A, B)$ as a function t with $\Delta = -1$ and $\Omega = 0.2$. The system is occupied by UNI phase. The corresponding phase space trajectories are plotted in panel (a2). The system starts from red dot and decay to the fixed point (blue dot). (a3) shows population difference $\tilde{s}_z = s_z^A - s_z^B$ and the average Rydberg population n_r. For UNI phase, $\tilde{s}_z = 0$. (b1) Evolution of n_r^j with $\Delta = -1$ and $\Omega = 0.9$. The system is in the OSC phase. Their phase space trajectories begin to oscillate persistently, and give rise to limit cycles [see panel (b2)]. (b3) $\tilde{s}_z$ and n_r are also non-stationary. (c1) The system is in the AF phase when increasing Ω to 2.8. (c2) The phase space trajectories decay to two different fixed points (blue dot). (c3) The order parameter $\tilde{s}_z$ is non-zero in AF phase. The panel (a), (b) and (c) correspond to points A, B, and C in Fig. S2(b).

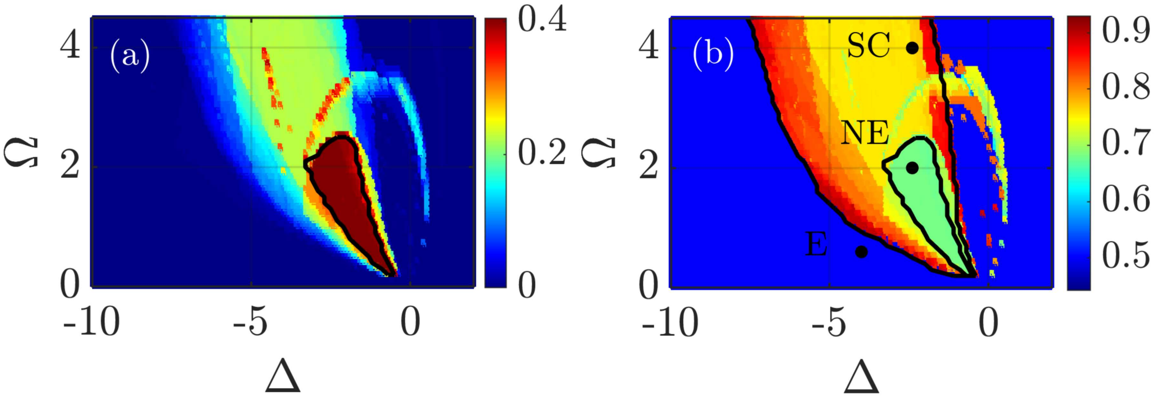

Figure S4: DTWA phase diagram and Hopkins statistic. We use the different order parameters to sketch the DTWA phase diagram in $\Omega - \Delta$ space. (a) The ratio N_{active}/N. (b) The Hopkins H is calculated to identify the formation of Rydberg clusters, where $H \simeq 0.5$ indicates homogeneous distribution and $H > 0.5$ means Rydberg clusters form. Based on the magnitude of H, the system can be classified into stationary without clusters (E-region), oscillatory with clusters (NE-region), and stationary with cluster (SC-region) phases.

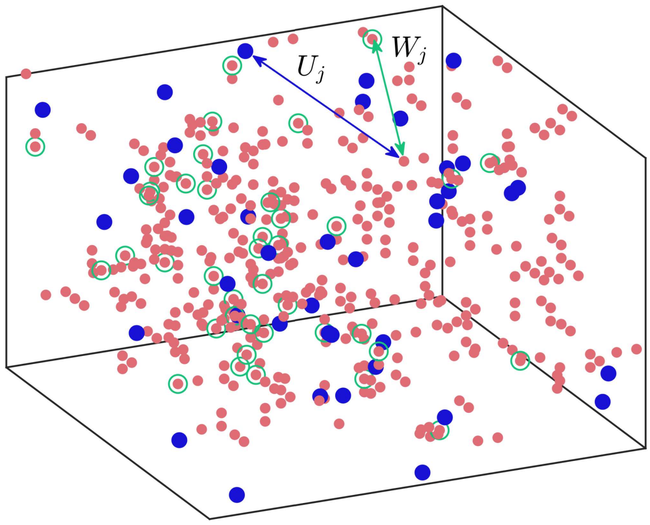

Figure S5: The Hopkins statistics of active atoms. M test points is sampled randomly [i.e., $\mathbf{X}_t = (x_j^t, y_j^t, z_j^t)$, $j = 1, 2, ..M$] in our simulation space (see blue dots); The position of active atom can be identified with $\mathbf{X}_a = (x_j^a, y_j^a, z_j^a)$, ($j = 1, 2, ..N_{\text{active}}$) (see red dots). Two types of distances can be defined with (i) the distance U_j is found from $\mathbf{X}_t$ to its nearest site in active ensemble $\mathbf{X}_a$; (ii) the distance W_j is from a randomly selected site in $\mathbf{X}_a$ to its nearest neighbor (M out of the available N_{active} sites are marked with green circle).

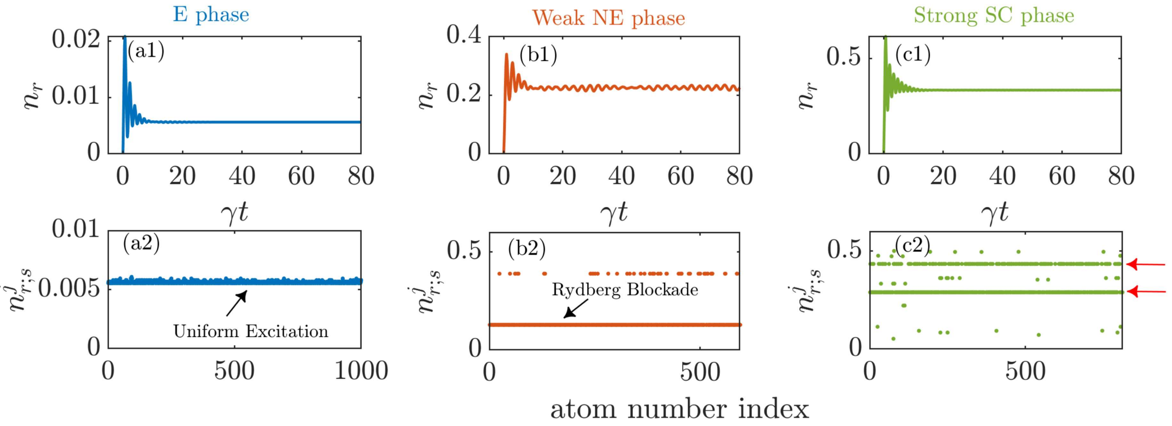

Figure S6: **Dynamics of the atom ensemble.** (a1) mean values of the Rydberg population n_r as a function t with $\Omega = 0.6$ and $\Delta = -4$. The system is in the E-phase. We plot fixed points of corresponding inactive atoms in panel (a2). All fixed points are identical (up to a small fluctuation). (b1) n_r^j varies with t for $\Omega = 2$ and $\Delta = -2.4$. The system is in the NE regime. In panel (b2), we can find that most of inactive atoms have very small Rydberg populations. (c1)-(c2) The system is in the SC phase when increasing Ω to 4. At later time, the average Rydberg population is stationary. Different atoms occupy very different Rydberg populations. Here a large fraction of the fixed points decay to two different values (highlighted by two red arrows). The panel (a), (b) and (c) correspond to E-, NE-, and SC- phases in Fig. S4(c).

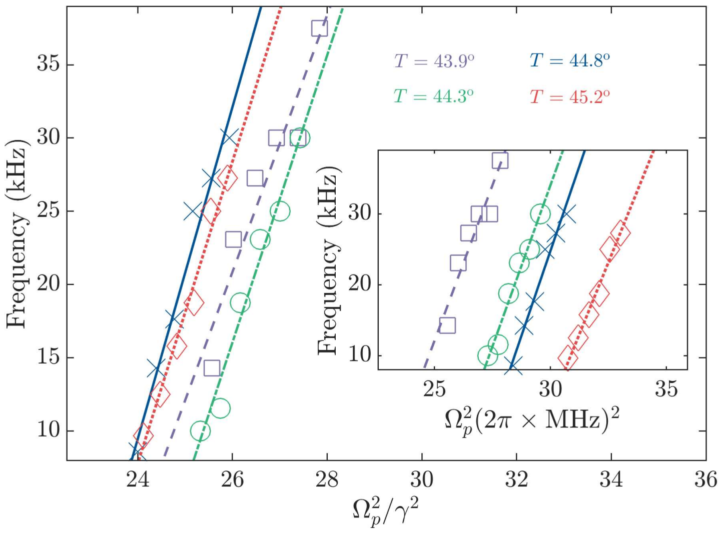

Figure S7: **The the oscillation frequency.** Oscillation frequencies as a function of the scaled probe field power. The oscillation frequency increases with the power of probe field monotonically. The inset shows the dependence of the frequency on the temperature of the atomic gas. When scaled with the decay rate, these curves almost collapse.

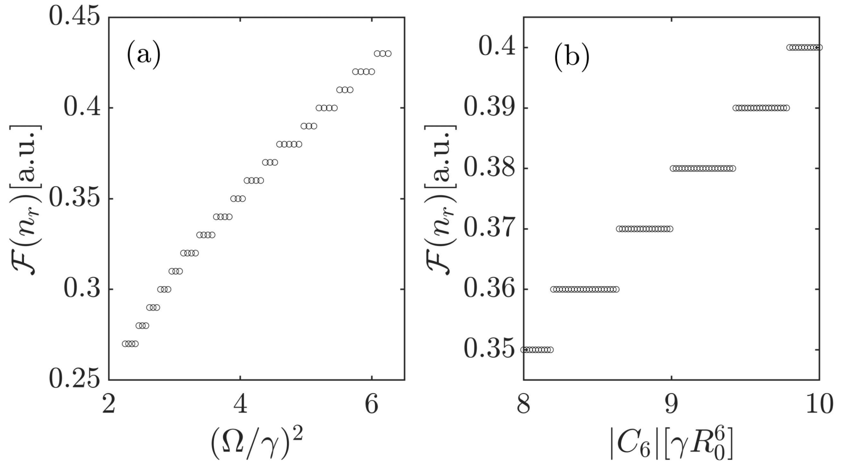

Figure S8: **The oscillation frequency obtained from the DTWA simulation.** Oscillation frequencies varies with (a) driving field Ω^2 and (b) interaction strength C_6. This dependence with Ω^2 agrees with our experimental results shown in Figure S7.

REFERENCES AND NOTES

1. M. Srednicki, Chaos and quantum thermalization. *Phys. Rev. E* **50**, 888–901 (1994).
2. M. Srednicki, The approach to thermal equilibrium in quantized chaotic systems. *J. Phys. A. Math. Gen.* **32**, 1163–1175 (1999).
3. L. E. Reichl, *A Modern Course in Statistical Physics* (Wiley-VCH, 2016).
4. L. Boltzmann, Ueber die mechanischen analogien des zweiten hauptsatzes der thermodynamik. *J. für die Reine und Angew. Math.* **1887**, 201–212, (1887).
5. G. Venkataraman, D. Sahoo, V. Balakrishnan, *Beyond the Crystalline State: An Emerging Perspective* (Springer-Verlag, 1989).
6. T. Kinoshita, T. Wenger, D. S. Weiss, A quantum newton's cradle. *Nature* **440**, 900–903 (2006).
7. R. J. Baxter, *Exactly Solved Models in Statistical Mechanics* (Dover Publications Inc., 2008).
8. R. Nandkishore, D. A. Huse. Many-body localization and thermalization in quantum statistical mechanics. *Annu. Rev. Condens. Matter Phys.* **6**, 15–38 (2015).
9. D. A. Abanin, E. Altman, I. Bloch, M. Serbyn. Colloquium: Many-body localization, thermalization, and entanglement. *Rev. Mod. Phys.* **91**, 021001 (2019).
10. C. J. Turner, A. A. Michailidis, D. A. Abanin, M. Serbyn, Z. Papić, Weak ergodicity breaking from quantum many-body scars. *Nat. Phys.* **14**, 745–749 (2018).
11. A. Browaeys, T. Lahaye, Many-body physics with individually controlled Rydberg atoms. *Nat. Phys.* **16**, 132–142 (2020).
12. H. Labuhn, D. Barredo, S. Ravets, S. De Léséleuc, T. Macrì, T. Lahaye, A. Browaeys, Tunable two-dimensional arrays of single Rydberg atoms for realizing quantum ising models. *Nature* **534**, 667–670 (2016).
13. H. Zhao, A. Smith, F. Mintert, J. Knolle, Orthogonal quantum many-body scars. *Phys. Rev. Lett.* **127**, 150601 (2021).
14. S. Choi, C. J. Turner, H. Pichler, W. W. Ho, A. A. Michailidis, Z. Papić, M. Serbyn, M. D. Lukin, D. A. Abanin, Emergent SU (2) dynamics and perfect quantum many-body scars. *Phys. Rev. Lett.* **122**, 220603 (2019).
15. M. D. Lukin, M. Fleischhauer, R. Cote, L. M. Duan, D. Jaksch, J. I. Cirac, P. Zoller, Dipole blockade and quantum information processing in mesoscopic atomic ensembles. *Phys. Rev. Lett.*, **87**, 037901 (2001).

16. P. Schauß, M. Cheneau, M. Endres, T. Fukuhara, S. Hild, A. Omran, T. Pohl, C. Gross, S. Kuhr, I. Bloch, Observation of spatially ordered structures in a two-dimensional Rydberg gas. *Nature* **491**, 87–91 (2012).
17. H. Bernien, S. Schwartz, A. Keesling, H. Levine, A. Omran, H. Pichler, S. Choi, A. S. Zibrov, M. Endres, M. Greiner, V. Vuletić, M. D. Lukin, Probing many-body dynamics on a 51-atom quantum simulator. *Nature* **551**, 579–584 (2017).
18. D. Bluvstein, A. Omran, H. Levine, A. Keesling, G. Semeghini, S. Ebadi, T. T. Wang, A. A. Michailidis, N. Maskara, W. W. Ho, S. Choi, M. Serbyn, M. Greiner, V. Vuletić, and M. D. Lukin, Controlling quantum many-body dynamics in driven Rydberg atom arrays. *Science* **371**, 1355–1359 (2021).
19. T. E. Lee, H. Häffner, M. C. Cross, Collective quantum jumps of Rydberg atoms. *Phys. Rev. Lett.* **108**, 023602 (2012).
20. T. E. Lee, H. Häffner, M. C. Cross, Antiferromagnetic phase transition in a nonequilibrium lattice of Rydberg atoms. *Phys. Rev. A* **84**, 031402(R) (2011).
21. I. Lesanovsky, Liquid ground state, gap, and excited states of a strongly correlated spin chain. *Phys. Rev. Lett.* **108**, 105301 (2012).
22. J. Qian, L. Zhou, W. Zhang, Quantum phases of strongly interacting Rydberg atoms in triangular lattices. *Phys. Rev. A* **87**, 063421 (2013).
23. M. Marcuzzi, E. Levi, S. Diehl, J. P. Garrahan, I. Lesanovsky, Universal nonequilibrium properties of dissipative Rydberg gases. *Phys. Rev. Lett.* **113**, 210401 (2014).
24. N. Malossi, M. M. Valado, S. Scotto, P. Huillery, P. Pillet, D. Ciampini, E. Arimondo, O. Morsch, Full counting statistics and phase diagram of a dissipative rydberg gas. *Phys. Rev. Lett.* **113**, 023006 (2014).
25. H. Weimer, Variational principle for steady states of dissipative quantum many-body systems. *Phys. Rev. Lett.* **114**, 040402 (2015).
26. E. Levi, R. Gutiérrez, I. Lesanovsky Quantum non-equilibrium dynamics of Rydberg gases in the presence of dephasing noise of different strengths. *J. Phys. B At. Mol. Opt. Phys.* **49**, 184003 (2016).
27. R. Gutiérrez, C. Simonelli, M. Archimi, F. Castellucci, E. Arimondo, D. Ciampini, M. Marcuzzi, I. Lesanovsky, O. Morsch, Experimental signatures of an absorbing-state phase transition in an open driven many-body quantum system. *Phys. Rev. A* **96**, 041602(R) (2017).

28. A. Cabot, L. S. Muhle, F. Carollo, I. Lesanovsky, *Phys. Rev. A* Quantum trajectories of dissipative time-crystals. **108**, L041303 (2023).
29. C. Carr, R. Ritter, C. G. Wade, C. S. Adams, K. J. Weatherill, Nonequilibrium phase transition in a dilute Rydberg ensemble. *Phys. Rev. Lett.* **111**, 113901 (2013).
30. D.-S. Ding, H. Busche, B.-S. Shi, G.-C. Guo, C. S. Adams, Phase diagram and self-organizing dynamics in a thermal ensemble of strongly interacting Rydberg atoms. *Phys. Rev. X* **10**, 021023 (2020).
31. S. Helmrich, A. Arias, G. Lochead, T. M. Wintermantel, M. Buchhold, S. Diehl, S. Whitlock, Signatures of self-organized criticality in an ultracold atomic gas. *Nature* **577**, 481–486 (2020).
32. M. Gärttner, K. P. Heeg, T. Gasenzer, J. Evers, Dynamic formation of Rydberg aggregates at off-resonant excitation. *Phys. Rev. A* **88**, 043410 (2013).
33. I. Lesanovsky, J. P. Garrahan, Out-of-equilibrium structures in strongly interacting Rydberg gases with dissipation. *Phys. Rev. A* **90**, 011603(R) (2014).
34. A. Urvoy, F. Ripka, I. Lesanovsky, D. Booth, J. P. Shaffer, T. Pfau, R. Löw, Strongly correlated growth of rydberg aggregates in a vapor cell. *Phys. Rev. Lett.* **114**, 203002 (2015).
35. F. Letscher, O. Thomas, T. Niederprüm, M. Fleischhauer, H. Ott, Bistability versus metastability in driven dissipative rydberg gases. *Phys. Rev. X* **7**, 021020 (2017).
36. M. Tanasittikosol, C. Carr, C. S. Adams, K. J. Weatherill, Subnatural linewidths in two-photon excited-state spectroscopy. *Phys. Rev. A* **85**, 033830 (2012).
37. R. M. Potvliege, C. S. Adams, Photo-ionization in far-off-resonance optical lattices. *New J. Phys.* **8**, 163 (2006).
38. S. Geier, N. Thaicharoen, C. Hainaut, T. Franz, A. Salzinger, A. Tebben, D. Grimshandl, G. Zürn, M. Weidemüller, Floquet hamiltonian engineering of an isolated many-body spin system. *Science* **374**, 1149–1152 (2021).
39. J. Schachenmayer, A. Pikovski, A.M. Rey Many-body quantum spin dynamics with Monte Carlo trajectories on a discrete phase space. *Phys. Rev. X* **5**, 011022 (2015).
40. V. P. Singh, H. Weimer, Driven-dissipative criticality within the discrete truncated wigner approximation. *Phys. Rev. Lett.* **128**, 200602 (2022).
41. M. Marcuzzi, J. Minář, D. Barredo, S. de Léséleuc, H. Labuhn, T. Lahaye, A. Browaeys, E. Levi, I. Lesanovsky, Facilitation dynamics and localization phenomena in Rydberg lattice gases with position disorder. *Phys. Rev. Lett.* **118**, 063606 (2017).

42. T. M. Wintermantel, M. Buchhold, S. Shevate, M. Morgado, Y. Wang, G. Lochead, S. Diehl, S. Whitlock, Epidemic growth and Griffiths effects on an emergent network of excited atoms. *Nat. Commun.* **12**, 103 (2021).
43. A. Banerjee, R.N. Dave, Validating clusters using the hopkins statistic, in *2004 IEEE International Conference on Fuzzy Systems* (IEEE, catalog no. 04CH37542, 2004).
44. H. Schempp, G. Günter, M. Robert-de Saint-Vincent, C. S. Hofmann, D. Breyel, A. Komnik, D. W. Schönleber, M. Gärttner, J. Evers, S. Whitlock, M. Weidemüller. Full counting statistics of laser excited rydberg aggregates in a one-dimensional geometry. *Phys. Rev. Lett.* **112**, 013002 (2014).
45. M. Serbyn, D. A. Abanin, Z. Papić. Quantum many-body scars and weak breaking of ergodicity. *Nat. Phys.* **17**, 675–685 (2021).
46. A. Keesling, A. Omran, H. Levine, H. Bernien, H. Pichler, S. Choi, R. Samajdar, S. Schwartz, P. Silvi, S. Sachdev, P. Zoller, M. Endres, M. Greiner, V. Vuletić, M. D. Lukin. Quantum kibble–zurek mechanism and critical dynamics on a programmable rydberg simulator. *Nature* **568**, 207–211 (2019).
47. B. Buča, J. Tindall, D. Jaksch. Non-stationary coherent quantum many-body dynamics through dissipation. *Nat. Commun.* **10**, 1730 (2019).
48. F. Ripka, H. Kübler, R. Löw, T. Pfau, A room-temperature single-photon source based on strongly interacting Rydberg atoms. *Science* **362**, 446–449 (2018).
49. F. Verstraete, M. M. Wolf, J. Ignacio Cirac. Quantum computation and quantum-state engineering driven by dissipation. *Nat. Phys.* **5**, 633–636 (2009).
50. S. Diehl, A. Tomadin, A. Micheli, R. Fazio, P. Zoller, Dynamical phase transitions and instabilities in open atomic many-body systems. *Phys. Rev. Lett.*, **105**, 015702 (2010).
51. S. H. Strogatz. *Nonlinear Dynamics and Chaos: With Applications to Physics, Biology, Chemistry, and Engineering* (CRC Press, ed. 2, 2015).
52. A. Gallagher, E. L. Lewis, Determination of the vapor pressure of rubidium by optical absorption. *J. Opt. Soc. Am.* **63**, 864–869 (1973).
